# Modeling the Spread of Methicillin-Resistant *Staphylococcus aureus* in Nursing Homes for Elderly

**DOI:** 10.1371/journal.pone.0029757

**Published:** 2012-01-06

**Authors:** Farida Chamchod, Shigui Ruan

**Affiliations:** Department of Mathematics, University of Miami, Coral Gables, Florida, United States of America; Massey University, New Zealand

## Abstract

Methicillin-resistant *Staphylococcus aureus* (MRSA) is endemic in many hospital settings, including nursing homes. It is an important nosocomial pathogen that causes mortality and an economic burden to patients, hospitals, and the community. The epidemiology of the bacteria in nursing homes is both hospital- and community-like. Transmission occurs via hands of health care workers (HCWs) and direct contacts among residents during social activities. In this work, mathematical modeling in both deterministic and stochastic frameworks is used to study dissemination of MRSA among residents and HCWs, persistence and prevalence of MRSA in a population, and possible means of controlling the spread of this pathogen in nursing homes. The model predicts that: 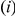 without strict screening and decolonization of colonized individuals at admission, MRSA may persist; 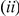 decolonization of colonized residents, improving hand hygiene in both residents and HCWs, reducing the duration of contamination of HCWs, and decreasing the resident∶staff ratio are possible control strategies; 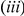 the mean time that a resident remains susceptible since admission may be prolonged by screening and decolonization treatment in colonized individuals; 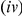 in the stochastic framework, the total number of colonized residents varies and may increase when the admission of colonized residents, the duration of colonization, the average number of contacts among residents, or the average number of contacts that each resident requires from HCWs increases; 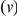 an introduction of a colonized individual into an MRSA-free nursing home has a much higher probability of leading to a major outbreak taking off than an introduction of a contaminated HCW.

## Introduction

Methicillin-resistant *Staphylococcus aureus* (MRSA) has been recognized as a major nosocomial pathogen responsible for morbidity and mortality in hospitals and other healthcare settings globally [1], [2]. The pathogen causes a wide range of syndromes: skin and soft tissue infections, bloodstream infection, and pneumonia, for instance. Colonization of MRSA may take place in many parts of the body: axillae, perineum, groin, rectum, skin, and anterior nares. It has been suggested that individuals who are persistently colonized with MRSA have a greater propensity to develop infection than uncolonized and short-term colonized individuals [3]. Infections with MRSA were reported two years after using methicillin to treat individuals infected with penicillin-resistant *S. aureus* [4]. MRSA has since become endemic in hospitals and healthcare settings globally [5], [6].

Nursing homes are known as skilled nursing facilities for seniors who generally require constant medical care and significant assistance in daily living. Residents are normally under the care of registered nursing staff or nursing assistants. Most residents are likely to have chronic and multiple diseases. Several studies have shown that MRSA colonization increases with advancing age and is highest in those over 70 years old [7], [8]. As high demand for hospitalizations in some hospitals often results in a shorter length of stay of many patients, large numbers of patients colonized with MRSA are discharged to nursing homes. Consequently, residents in nursing homes have a tendency to serve as a reservoir of MRSA. Also, it is quite common that some residents are readmitted into hospitals and can be unintentional vectors disseminating the pathogens between hospitals and nursing homes. MRSA colonization has been shown to be associated with higher mortality to residents in nursing homes and those who are persistently colonized have a greater risk at developing infections [9]–[11]. Risk factors of colonization among residents include: hospitalization, exposure to antibiotics, low nursing staff∶residents ratios, and contact activities. Transmission of MRSA from resident to resident occurs via contaminated hands of health-care workers (HCWs), direct contacts among residents, and indirect contacts via shared objects. In addition, it has been shown that MRSA in healthcare settings can be isolated from skin surfaces and hands of HCWs (transiently) [12]–[14]. The epidemiology of hospital-acquired bacteria differs from community-acquired bacteria in many aspects: large daily influx and efflux, opportunistic infection, a high rate of antibiotic usage, and comorbidity with other diseases, for instance. Another difference from hospitals is that residents in nursing homes tend to stay in the facilities longer and participate in some social activities.

Mathematical modeling has been significantly used to understand the spread of nosocomial pathogens in hospital settings [15]–[26]. In particular, mathematical models based on vector-borne diseases were developed to investigate transmission dynamics among patients via hands of HCWs and persistence of MRSA [16], [17] and vancomycin-resistant enterococci in hospital settings [18], [20], [24]. So far, only a few studies have specifically considered the transmission dynamics and control strategies of MRSA in nursing homes, although it has been repeatedly shown that MRSA is highly endemic in many nursing homes worldwide and nursing homes may pay a crucial role in spreading MRSA to the hospitals and community. For example, a mathematical model was developed to investigate dynamics of antibiotic-resistant bacteria (ARB) between hospitals and long-term care facilities [22]. In this article, however, we focus on the transmission dynamics of MRSA inside nursing homes and differentiate between two modes of transmission, transmission via HCWs and transmission among residents. We use a simple mathematical model to describe transmission dynamics of MRSA in nursing homes. The model considers the changes in the populations of uncolonized and colonized residents, and uncontaminated and contaminated HCWs. In particular, we aim to understand persistence and prevalence of MRSA, and possible means to control MRSA in nursing homes using both deterministic and stochastic frameworks. Note that the former simply provides biological understanding of the disease dissemination while the latter takes into account random effects that result in variability of results.

## Methods

**Figure 1:**
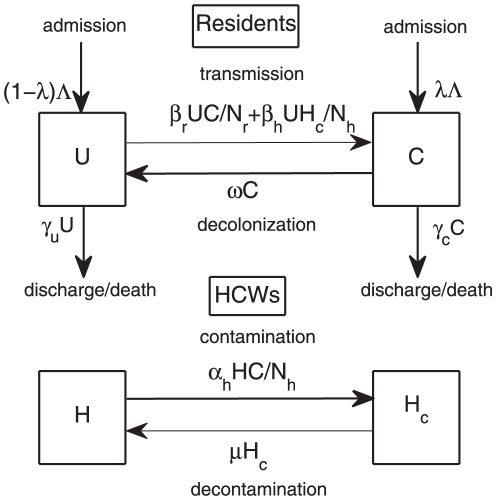
Diagram. A compartment model to describe transmission dynamics of MRSA in nursing homes. The diagram shows the inflow and outflow of uncolonized and colonized residents 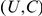, and uncontaminated and contaminated HCWs 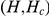.

We use mathematical models to study the transmission dynamics of MRSA among residents in nursing homes. HCWs play an important role in transmitting MRSA from patient-to-patient in both hospitals and nursing homes [14]. They disseminate MRSA but are assumed not to develop clinical MRSA infections. Hence, a framework for vector-borne diseases and frequency-dependent transmission is employed [27]. Here, we differentiate transmission of MRSA in nursing homes from hospitals by taking into account not only contacts between HCWs and residents, but also contacts among residents themselves during social activities. Based on the framework for vector-borne diseases, HCWs are viewed as transient vectors and residents as definite hosts [18], [20], [24]. Residents are divided into two groups: uncolonized and colonized with MRSA (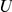 and 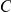). We do not distinguish between colonized and clinically infected residents, because in general, being colonized with MRSA may put residents at risk of developing infections that may lead to mortality, morbidity and co-morbidity with other diseases, and residents are likely to be transferred to hospitals due to infections. HCWs are separated into two groups: uncontaminated and contaminated with MRSA (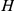 and 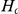). A flow diagram is depicted in Figure 1. The governing system of equations is described by
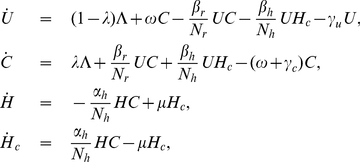
(1)Details for parameters in this model can be found in Table 1. The populations of patients and HCWs are considered to be homogenous. It is assumed that the number of residents and HCWs remains fixed (reflecting the limited resources) and bed occupancy is 100%. Hence, the admission rate equals the discharge rate (i.e., 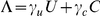). Note that the discharge rate takes into account both normal discharges and deaths of residents. In the model, the probability that an individual is colonized at admission is 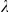. Decolonization of residents by treatment or clearance and decontamination of HCWs by hand washing occur at rate 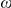 and 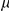, respectively. Here, we ignore transmission caused by long-term staff carriers and only consider transmission caused by transiently colonized HCWs. We define 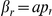 as the resident-resident transmission rate, 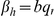 as the HCW-resident transmission rate, and 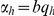 as the resident-HCW transmission rate. Instead of assuming that each HCW contacts residents with a constant rate (in a way similar to mosquitoes biting humans, so that humans are bitten proportionely to the number of mosquitoes), we made the slightly different assumption that each resident requires a number of contacts from HCWs per day. Based on the framework for vector-borne diseases, we assume that the average number of contacts that each resident requires from HCWs per day 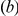 is constant and it is shared among HCWs so that the rate at which a particular HCW contacts a particular resident is 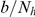, where 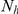 is the total number of HCWs. By this assumption, HCW-to-resident transmission depends on HCWs, and the rate at which HCWs contact residents increases in proportion to the number (or density) of residents. We assume that a susceptible resident becomes colonized during contact with a contaminated HCW with the probability 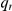 and a HCW becomes contaminated by contact with a colonized resident with the probability 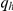. In a similar way, for consistency of the system, we assume that contacts among residents are fixed and shared among residents for resident-to-resident transmission, so that each resident contacts other residents with a constant rate. Hence, a particular resident contacts another resident at rate 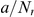, where 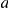 is the average number of contacts made by each resident and 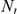 is the total number of residents. An uncolonized resident becomes colonized during contact with a colonized resident with the probability 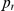. It is assumed that contamination in HCWs is removed at rate 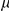 by hand washing.

**Table 1:** Parameters for the models.

Description	Symbol	Value	References
The total number of residents	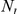	2000	estimated from 3–4 nursing
			homes in the community
Number of residents per number of HCWs	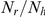	3,4	[35], [36]
Probability of admission of colonized residents	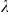	0.1	[37]
			[38]
Average duration of colonization (days)	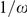	60,80	[39]
Average length of stay of uncolonized residents (days)	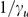	365	[40]
Average length of stay of colonized residents (days)	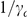	365	[40]
Resident-resident transmission rate	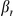	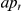	
HCW-resident transmission rate	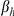	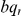	
Resident-HCW transmission rate	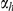	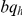	
Average number of contacts between residents	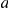	1	estimated
Probability of colonization via contacts of residents	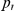	0.015	[41]
Probability of colonization via contacts of HCWs	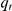	0.015	[26], [41]
Probability of contamination of HCWs	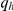	0.015	[41]
Average number of required contacts from HCWs	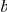	8	estimated
Average duration of contamination (hours)	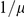	0.5,1	[18]

Both deterministic and stochastic models are studied. Both types of models play a crucial role in understanding the mechanisms of disease dissemination. The qualitative study of the deterministic model (which approximates the corresponding stochastic model) is an important and simple means to understand and gain information about disease dynamics and threshold behaviors. However, the deterministic model excludes randomness of events, and fractions of individuals can occupy a state rather than integers. In contrast to the deterministic model, the study of the stochastic model may not be simple but it demonstrates variability of results and considers fadeout effects that may be important if small numbers of individuals are initially colonized or infected. For the stochastic model, we use the continuous-time Markov chain process (CTMC) and an algorithm is based on the Gillespie's First Reaction algorithm for event-driven approaches [28]. Thanks to the limited number of beds in nursing homes, the process is bivariate, 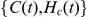 with 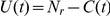 and 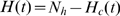. Hence, a joint probability function is described by
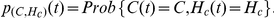
Transitions between classes are shown in Table 2.

**Table 2:** Transitions between classes.

Event	Transition	Probability of transition event occurs in 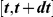
Admission of colonized residents	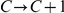	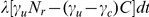
Transmission among residents	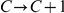	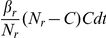
Transmission via HCWS	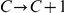	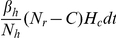
Decolonization	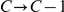	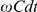
Death of uncolonized residents	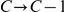	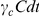
Contamination	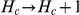	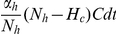
Decontamination	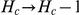	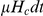

## Results

### Deterministic process

**Figure 2:**
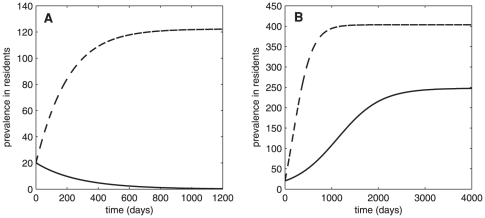
Persistence of MRSA. Time series data showing prevalence of MRSA colonization (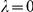-solid trace, 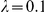-dash trace). (A) When 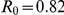 (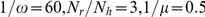), MRSA dies out if 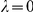 and is endemic in the population if 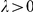 (B) When 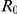 = 1.14 (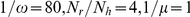), MRSA persists whether there is admission of colonized individuals or not.

Equilibrium quantities, a proof of their existence and uniqueness, and their stability conditions can be found in the electronic supplementary material, File S1. When colonized individuals constantly enter nursing homes 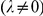, colonization of MRSA among residents always persists (only the disease-present steady state exists and is stable). In case there is no admission of colonized individuals (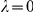) (this may happen when screening at admission is very strict), two possibilities can occur, either MRSA dying out or persisting in the population. MRSA dies out under the condition:
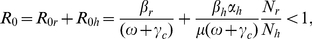
(2)where 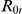 is the basic reproductive number excluding transmission via hands of HCWs and 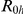 is the basic reproductive number when transmission among residents is omitted. MRSA persists if and only if 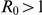. From this condition, persistence of MRSA depends on transmission of MRSA, the average length of stay of colonized residents, average time of colonization, hand hygiene in both residents and HCWs, and the resident∶staff ratio. Moreover, it is more likely to occur with higher rate of transmission, longer length of stay of colonized residents, longer time of colonization of residents, longer time of contamination in HCWs, and higher resident∶staff ratio. Figure 2 shows that the prevalence of MRSA in nursing homes approaches the endemic steady state when colonized individuals are constantly admitted to nursing homes. When there is no admission of colonized individuals, whether the prevalence of MRSA approaches the endemic steady state or the disease-free steady state depends on 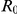 and the threshold value 1 (see also Figure 2). MRSA dies out if and only if 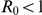 and persists if and only if 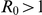.

**Figure 3:**
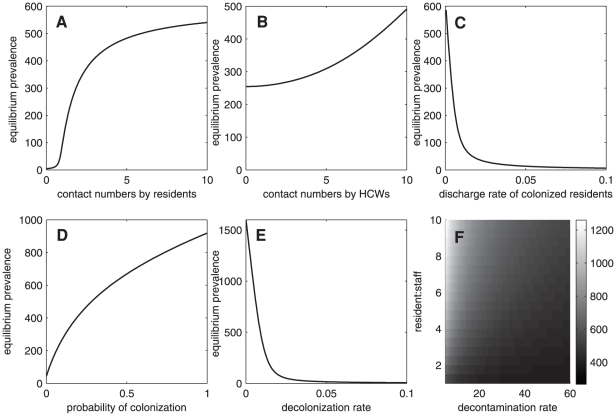
Prevalence of MRSA. Prevalence of MRSA in nursing homes at the steady states (from the deterministic model) as a function of the average number of contacts among residents (A), the average number of contacts that each resident requires (B), the discharge rate of colonized residents (C), probability of colonization at admission (D), rate of decolonization (E), and the decontamination rate and the resident∶staff ratio (F) (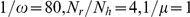).

Figure 3 shows the long-term prevalence of MRSA relating to various factors. We investigate the asymptotic solution of colonized residents 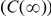 or equilibrium prevalence when some parameters vary. The prevalence of MRSA among residents increases when the number of contacts among residents increases 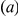 (see Figure 3A). As the prevalence increases rapidly before saturating, this suggests that the number of contacts among residents may be one of the important predictors of MRSA dissemination. Note that this result is associated with hand hygiene compliance of residents, and noncompliance and increasing number of contacts may lead to the higher prevalence of MRSA (see File S1). In Figure 3B, the prevalence of MRSA increases when the number of contacts that a resident requiring from HCWs per day increases 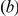 (this reflects the higher assumed rate of MRSA transmission via hands of HCWs). This result is associated with hand hygiene compliance of HCWs, and noncompliance may lead to the higher prevalence of MRSA. Moreover, in Figure 3C, the length of stay of colonized residents 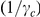 may escalate the prevalence of MRSA. However, this result may not be of much help in designing control strategies because whether residents stay longer or leave quickly also depends on other factors such as age or age-associated diseases. The model suggests that the higher the rate of admissions of colonized individuals 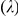, the higher will be the prevalence of MRSA in the population (see Figure 3D). Hence, to reduce the prevalence of MRSA, it may be worth considering screening and decolonization at admission. However, this method may not be cost-effective. The model also predicts that reducing colonization time in colonized residents helps to decrease the prevalence of MRSA drastically (see Figure 3E). Understaffing and prolonging the time between decontaminations of HCWs may lead to the higher number of colonized residents (see Figure 3F). Finally, understaffing and noncompliance of HCWs may increase the prevalence of MRSA in nursing homes (see File S1). Thus, increasing the number of HCWs and hand hygiene compliance may help to reduce MRSA transmission.

**Figure 4:**
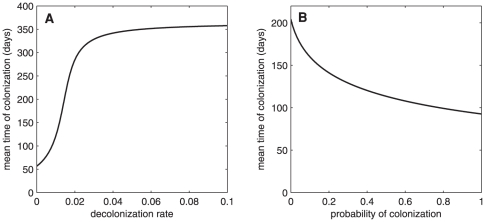
The mean time of colonization. The mean time of colonization since admission: (A) when the decolonization rate 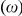 varies, (B) when probability of colonization at admission 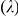 varies (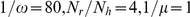).

We further investigate the mean time of colonization since admission or the mean time that a resident remains susceptible since admission [27]. It is given by:
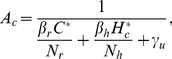
(3)where 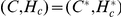 is the endemic steady state. We emphasize here that a lower rate of admissions of colonized individuals and a faster rate of decolonization may play important roles in lengthening the mean time to colonization since admission (see Figure 4). From this prediction, efficient screening process at admission and decolonization of colonized individuals may be two of the important keys in prolonging the susceptibility time of residents and, consequently, mitigating their co-morbidity of MRSA infection with other diseases.

### Stochastic process

Although the deterministic model has provided intensive biological understanding of the disease transmission dynamics, it is also important to take into account variability of results and fadeout effects in determining patterns of disease incidence and persistence [29]. Hence, we employ a Markov population process with continuous time and discrete state space to investigate the random effects.

The transition probabilities of this bivariate process are shown in Table 2. The forward Kolmogorov differential equations for the state probabilities, the moment generating function equation, the cumulant generating function equation, and the mean, variance, and covariance equations for 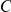 and 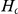 are fully discussed in File S1.

**Figure 5:**
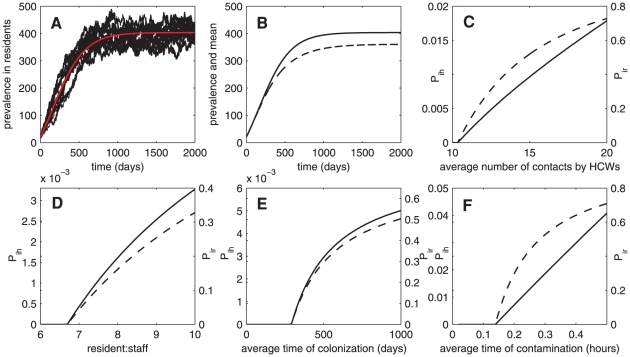
Stochasticity. (A) Time series for the deterministic and stochastic models showing the prevalence of MRSA colonization (examples of 10 from 5000 realizations)(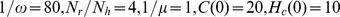). (B) Comparison between the deterministic time series and the mean solution from the equations of mean, variance, and covariance (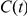-solid trace and 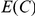-dash trace). (C)–(F) Invasion probability of MRSA according to the changes of the average number of contacts between residents and HCWs, the resident∶staff ratio, the period of contamination time in HCWs, and the period of colonization time in residents, respectively (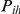-solid trace and 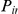-dash trace)(for 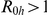, we set 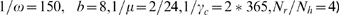).

Figure 5A shows twenty realizations of the time series of MRSA colonization among residents from the stochastic model and the corresponding result from the deterministic model. It indicates that population dynamics of colonized residents fluctuate over time in the stochastic framework. Moreover, the number of colonized residents in the limited time interval [0,2000] varies among twenty realizations (see Table 3). Stochastic results at various sizes of nursing homes can be found in File S1. We further investigate the total number of colonized residents in the limited time interval [0,2000] when certain parameters vary, and find that the total case number of colonized residents on the 2000th day increases when the probability of admission of colonized residents, the duration of colonization, the average number of contacts among residents, or the average number of contacts that each resident requires from HCWs increases (see Table 4).

**Table 3:** An example of case numbers of colonized residents at the end of time interval [0,2000] of 10 realizations.

	1	2	3	4	5
case number	397	372	340	443	330

	6	7	8	9	10
case number	364	384	373	335	385

**Table 4:** Average case numbers of colonized residents at the end of time interval [0,2000] according to changes in certain parameters (from 5000 realizations).

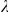	case number	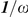	case number
0.0	138	1/60	157
0.1	375	1/80	375
0.2	503	1/100	619

Because the deterministic model is only an approximation of the stochastic model (by setting the second-order central moments equal to zero), it is not an exact representation of the mean behaviour of the system of finite populations [29]. Figure 5B demonstrates the difference between results from the deterministic and stochastic models, the number of colonized residents and its mean. Note that the mean value is obtained by solving the mean, variance, and covariance of the stochastic model (see File S1). It shows that the mean value of the number of colonized residents is lower than the time solution from the deterministic model. This result is due to the fact that extinction is taken into account in the stochastic model. The equilibrium values from the deterministic model are an approximation of the average behaviors of the stochastic model and can be obtained by assuming that the covariance is zero 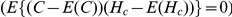 (but in general the covariance is non-zero [30]). Moreover, the mean values from the moment equations depend on the population size and the initial number of colonized residents and contaminated HCWs. The deterministic model is an approximation of the average behaviours of the stochastic model and can be obtained conditionally on no fade out.

In the stochastic framework, when the population size is very small, invasion of MRSA in an entirely susceptible population may not succeed, although 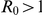, because stochastic extinction may take place after an introduction of sufficiently small numbers of colonized and contaminated individuals. If the population size and the initial number of colonized or contaminated individuals are sufficiently large, instead of an epidemic, MRSA may become endemic as it may take a long time until the epidemic ends. If 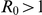 and the initial number of colonized or contaminated individuals is sufficiently small, there are two possibilities; either there is no epidemic or the size of the epidemic gets large and stays large for a long period of time. The probability that there is no epidemic (estimated from the probability of absorption) may relate to the basic reproductive number (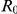) and the initial number of colonized and contaminated individuals. Note that in our stochastic model, MRSA may reemerge again after the extinction due to the presence of colonized residents at admission. Moreover, because two populations and two modes of transmission are involved, we do not consider this probability here. Instead, we investigate the invasion probability of MRSA when transmission among residents is omitted 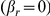. This is likely to occur in nursing homes for bed-bound residents with serious diseases or injuries or nursing homes in which residents have more private accommodations. The basic reproductive number of the system (1) with 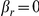 is
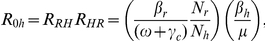
where 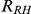 is the average number of residents directly infected by an introduction of a contaminated HCW into an entirely susceptible population of residents and 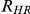 is the average number of HCWs directly infected by an introduction of a colonized resident into an entirely uncontaminated population of HCWs. In the stochastic model, invasion of MRSA in a susceptible population may not succeed although 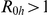, as stochastic extinction may instantly occur during the introduction of an infected individual. In this host-vector setting, we can calculate extinction and invasion probabilities by the use of multi-type branching processes [31]. When there are two types of populations (resident and HCW populations are labeled as 1 and 2, respectively), the distributions of secondary infections of each population can be described by the following generating functions:
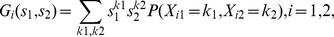
where 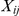 is the random variable that exhibits the number of secondary infection in population 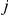 that arise from a single individual in population 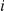. For 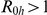, the invasion probability resulting from an introduction of a contaminated HCW is given by
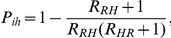
and the extinction probability is 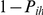 [30]. Similarly, the probability for a major outbreak to occur from an introduction of a colonized resident is
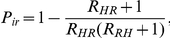
and the extinction probability is 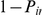. The invasion probability depends on many factors: the number of contacts, hand hygiene, decolonization time, and resident∶staff ratio. Both types of invasion probabilities increase according to the higher number of contacts that each resident requires per day and the higher resident∶staff ratio (see Figure 5C–D). In Figure 5E–F, both types of invasion probabilities decrease when lengths of colonization in residents and contamination in HCWs are shortened. Notice that the invasion probability of introducing a contaminated HCW into a nursing home is not greatly altered by the changes of parameters, while in contrast, the invasion probability of introducing a colonized resident is drastically changed by them. Based on the estimated duration of colonization and contamination, and neglecting long-term HCW carriers, the result suggests that an introduction of a colonized resident into an MRSA-free nursing home is more likely to lead to a major outbreak than an introduction of a contaminated HCW.

## Discussion

Although there has been considerable empirical study of MRSA colonization in nursing homes, detailed mathematical models based on this knowledge are still scant. We developed a mathematical model to study the transmission dynamics of MRSA in nursing homes. The model is based on the vector-borne disease framework, where hosts are residents and vectors are HCWs, and transmission of MRSA occurs via hands of HCWs transiently. Residents are separated into two groups: uncolonized and colonized, and HCWs are also separated into two groups: uncontaminated and contaminated. The epidemiology of the disease in nursing homes is similar to hospitals in many ways. However, there are still some differences: influx and efflux of residents in nursing homes are small comparing with hospitals; residents of nursing homes tend to stay in the facilities longer; and nursing homes are more community-like in that residents may share a room and involve in social activities. Hence, we took these factors into account in this work, particularly by including transmission among residents into the model. We studied the model in both deterministic and stochastic frameworks in an attempt to understand persistence and prevalence of MRSA, and sought possible ways to control the spread of MRSA in nursing homes. Both frameworks help to understand disease dynamics, threshold behaviors, possible control strategies, and variability of results.

In the deterministic framework, the model predicts that MRSA is always persistent in nursing homes when there is a constant influx of colonized individuals. Hence, it is possible that MRSA may be eliminated when there is no admission of colonized individuals. This result suggests that strict screening, decolonization by treatment, and isolation before admitting residents into nursing homes may be one of the first steps in starting the MRSA control program. However, although these strategies may be effective in controlling MRSA, they may not be cost-effective. From the model results, persistence and prevalence of MRSA depends on various factors: transmission rates, average length of stay of colonized residents, average time of colonization, hand hygiene, and resident∶staff ratio. Based on the model prediction, the prevalence of MRSA increases when the number of contacts among residents or the number of daily contacts that residents require from HCWs increases. Hence, if reducing social contacts among residents is not possible, our findings suggest that improving hand hygiene in residents may help to reduce the prevalence of MRSA in nursing homes. Our model results also suggest that understaffing and noncompliance of hand hygiene may lead to the higher prevalence of MRSA. Moreover, the model predicts that the prevalence of MRSA may be reduced by the shorter stay of colonized individuals and drastically reduced by the duration of colonization of MRSA, and the prevalence of MRSA may increase with more admission of colonized individuals. Reducing the number of colonized individuals at admission and the time of colonization have been shown to increase the mean time that a resident remains susceptible in our model results.

In the stochastic framework, we have taken into account randomness and fadeout effects that can lead to disease extinction and variability of results. A Markov population process with continuous time and discrete state space is used. Based on the stochastic model, we find that the total case number of colonized residents at the end of the limited time interval varies and increases when the probability of admission of colonized residents, duration of colonization, average number of contacts among residents, or average number of contacts that each resident requires from HCWs increases. Since the deterministic model is only an approximation of the stochastic model, the difference between the solutions of the deterministic model and the mean solution of the stochastic model is shown. Due to fadeout effects, MRSA may go extinct although the basic reproductive number of the system is greater than 1. The invasion probability, when transmission among residents is omitted, is further investigated. Our model predicts that, without taking into account the long-term MRSA carriage in HCWs, an introduction of a colonized individual into an MRSA-free nursing home is more likely to lead to a major outbreak than an introduction of a contaminated HCW into the facility. It also suggests that reducing the number of contacts between residents and HCWs, the resident∶staff ratio, the period of colonization time in residents, and the contamination time in HCWs may help to prevent a major outbreak in nursing homes.

Our study demonstrates that the number of contacts among residents, the number of contacts between residents and HCWs, admission of colonized residents, decolonization, decontamination, hand hygiene compliance and the length of stay of colonized residents in the facilities may be the most important predictors of the prevalence of MRSA in nursing homes. The prevalence of MRSA in nursing homes has been reported to be as high as 36% [32]. However, because the influx and efflux of residents in nursing homes are not as large as hospitals, interventions such as screening, isolation and decolonization may be some of the possible and efficient means to control the prevalence of MRSA, apart from improving hand hygiene in HCWs and residents. The model results from this work correspond to the previous studies [16] in that reducing the proportion of colonized individuals at admission is an effective way to control MRSA. Based on these findings, possible controls may include screening and isolation at admission.

In hospital intensive-care units, colonization of patients has been suggested to increase when the number of contacts between patients and HCWs increases [15]. This finding also corresponds with our model prediction in nursing homes. Because the number of contacts that each resident requires per day is based on necessity, it may not be reduced. Consequently, based on our findings that improving hand hygiene may help to reduce the prevalence of MRSA, possible controls may include hand washing with disinfecting agents and reducing contamination duration in HCWs. Moreover, in our model, we assumed that contact among residents is a factor in transmission and when this assumption was incorporated into the model, altering the contact rate among residents led to a difference in MRSA transmission. Under this assumption, our findings suggest that the higher number of contacts may lead to the higher prevalence of MRSA. Hence, if contact among residents is present, not only hand hygiene compliance in HCWs, but also hand hygiene compliance among residents may be important in the prevalence of MRSA in nursing homes.

Based on our simulations, the longer length of stay of colonized residents in nursing homes may increase the prevalence of MRSA. This result is consistent with some previous works [17], but is also in contrast with the prediction from some previous studies in hospitals [16] that decreasing length of stay of patients is more likely to result in outbreaks and the higher ward prevalence. Note that the findings of [16] are subtle, because reducing length of stay of patients brings more susceptibles into wards, and changes in length of stay have effects on the prevalence that depends on transmissibility due to stochastic effects. Also, note that whether residents stay in or leave nursing homes may depend on other factors such as age and age-associated disorders. Hence, reducing the length of stay of colonized residents may be impractical in the control program.

Our finding that the increase of the resident∶staff ratio may lead to the higher prevalence of MRSA is in contrast with some previous studies for hospitals [24] that the increase of the patient∶staff ratio results in the lower prevalence when the number of contacts is fixed according to the number of patients in our study and the number of HCWs in the previous studies. Based on our findings, increasing staff numbers and compliance of hand hygiene may help to reduce the prevalence of MRSA. Note that long-term staff carriers are not considered in this study. In hospitals, transmission from long-term staff carriers is rare, but it is not clear that this will be the case in nursing homes. Further investigation on how these HCWs influence the dynamics of MRSA in nursing homes remains challenging.

From the previous studies for hospitals [17], decolonization may not be effective in controlling MRSA, but our predictions suggest that decolonization of residents is an efficient way to control MRSA. The contradiction is possibly due to the longer length of stay of residents in nursing homes compared with the shorter length of stay of patients in hospitals. In comparison to MRSA, decolonization of individuals colonized with vancomycin-resistant *Enterococcus* and *Clostridium difficile* is not clinically suggested, because both of these can survive on environmental surfaces for long periods of time [33], [34]. Hence, strategies for controlling their prevalence in nursing homes may be slightly different from MRSA and should be further investigated.

In summary, our study suggests that possible strategies to control MRSA in nursing homes include screening at admission, decolonization of colonized residents, improving hand hygiene in residents and HCWs, and decreasing the resident∶staff ratio.

## Supporting Information

File S1 **The analysis of the deterministic model and the derivation of the stochastic model is given in the online supporting information file.**
(PDF)
