# Supplementary material for: Modeling the Spread of Methicillin-Resistant Staphylococcus aureus in Nursing Homes for Elderly
Source: PLoS One. 2012 Jan 6;7(1):e29757. doi: 10.1371/journal.pone.0029757 (PMC3253090; doi:10.1371/journal.pone.0029757)
Supplement: File S1 — The analysis of the deterministic model and the derivation of the stochastic model is given in the online supporting information file. (PDF) [file pone.0029757.s001.pdf]

Online Supporting Information
Modeling the spread of methicillin-resistant *Staphylococcus aureus* in nursing homes for elderly

By Farida Chamchod and Shigui Ruan

1 Analysis of the deterministic model

Because the number of beds in nursery homes is fixed, we assume that

$$\Lambda = \gamma_u U + \gamma_c C.$$

Hence, the total number of residents remains constant in the model. Note that the total number of HCWs is also constant.

1.1 Existence and Uniqueness of Steady states

- There exists a disease-free steady state only when there is no admission of colonized individuals ($\lambda = 0$). The disease-free steady state is as follows:

  $$(U, C, H, H_c) = (N_r, 0, N_h, 0).$$

  By setting the RHS of (1) in the main text equaling zeros, the disease-present steady state is as follows:

  $$(U, C, H, H_c) = \left(N_r - C^*, C^*, N_h - H_c^*, \frac{\alpha_h N_h C^*}{\mu N_h + \alpha_h C^*}\right),$$

  where $P(C^*)$ is a polynomial function of C^* and C^* satisfies the following equation:

  $$P(C^*) = c_3 C^{*3} + c_2 C^{*2} + c_1 C^* + c_0 = 0,$$

  with

  $$\begin{aligned} c_3 &= \beta_r \alpha_h, \\ c_2 &= \lambda \alpha_h N_r (\gamma_u - \gamma_c) + (\omega + \gamma_c)\alpha_h N_r + \beta_r \mu N_h + (\beta_h - \beta_r)\alpha_h N_r, \\ c_1 &= -\lambda \gamma_u \alpha_h N_r^2 + \lambda \mu N_r N_h (\gamma_u - \gamma_c) - \beta_r \mu N_r N_h - \beta_h \alpha_h N_r^2 + \mu N_r N_h (\omega + \gamma_c), \\ c_0 &= -\lambda \gamma_u \mu N_r^2 N_h. \end{aligned}$$

It is clear that c_0 is always negative and c_3 is always positive. We study the existence and uniqueness of the disease-present steady state by considering roots of $P(C^*)$ or using Descarte's rule of signs. We consider four cases of λ and β_r according to whether there are admission of colonized residents and contacts among residents.

1. $\lambda > 0$ and $\beta_r > 0$. By further assuming that $\beta_h > \beta_r$, it is obvious that c_2 is positive for all positive values of parameters (as $(1-\lambda)\alpha_h N_r \gamma_c > 0$). We rewrite the polynomial for C^* as follows:
$$P(C^*) = C_3 C^{*3} + C_2 C^{*2} \pm C_1 C^* - C_0,\ C_i > 0, i = 0, 1, 2, 3.$$
Either c_1 is positive or negative, there is only one sign change in $P(C^*)$ (+,+,+,-, or +,+,-,-). By Descarte's rule, there is one positive real root of $P(C^*)$. Set $\kappa = -C^*$. Hence,
$$P(\kappa) = C_3\kappa^3 - C_2\kappa^2 \pm C_1\kappa + C_0.$$
Whether c_1 is positive or negative, there are two sign changes in $P(\kappa)$ (+,-,+,+, or +,-,-,+). By Descarte's rule, there are either two or zero positive real roots of $P(\kappa)$ or in other words two or zero negative real roots of $P(C^*)$. Therefore, there are exactly one positive real root and possibly two negative real roots or complex conjugate roots of $P(C^*)$. In case $\beta_h > \beta_r$ is not assumed, it is possible that c_2 can be either positive or negative. If it is positive, it follows from the above result we have just shown. If not, let $c_2 = -C_2$. Hence,
$$\lambda\mu N_r N_h(\gamma_u - \gamma_c) + \mu N_r N_h(\omega + \gamma_c) = -\frac{\mu N_h C_2}{\alpha_h} - \frac{\beta_r \mu^2 N_h^2}{\alpha_h} - \beta_h \mu N_r N_h + \beta_r \mu N_r N_h.$$
Substituting this argument into c_1, we obtain
$$c_1 = -\lambda\gamma_u\alpha_h N_r^2 - \beta_h\alpha_h N_r^2 - \frac{\mu N_h C_2}{\alpha_h} - \frac{\beta_r\mu^2 N_h^2}{\alpha_h} - \beta_h\mu N_r N_h = -C_1.$$
Consequently, if c_2 is negative, we find that c_1 is also negative. By considering the change of signs, there is only one sign change (+,-,-,-) in $P(C^*)$ and there are two sign changes in $P(\kappa)$. Hence, there are only one positive real root and possibly two negative real roots or complex conjugate roots of $P(C^*)$. In conclusion, there always exists a unique non-zero positive real root C^* for all $\lambda > 0$ and $\beta_r > 0$.

2. $\lambda > 0$ and $\beta_r = 0$. This is likely to occur in nursery homes with bed-bound residents or private houses. We have $c_3 = 0$ in this case. Clearly, the coefficient c_2 is positive. We do not have information about the sign of c_1. Hence, we consider both positive and negative values of c_1. We write the polynomial $P(C^*)$ as follows:
$$P(C^*) = C_2 C^{*2} \pm C_1 C^* - C_0,\ C_i > 0, i = 0, 1, 2.$$
The explicit real solutions of $P(C^*)$ are
$$C^* = \frac{\mp C_1 \pm \sqrt{C_1^2 + 4C_0C_2}}{2C_2}.$$
Because $C_1 < \sqrt{C_1^2 + 4C_0C_2}$, we can always find a positive real root of $P(C^*)$ such that
$$C^* = \frac{-C_1 + \sqrt{C_1^2 + 4C_0C_2}}{2C_2} \text{ if } c_1 > 0 \text{ or } \frac{C_1 + \sqrt{C_1^2 + 4C_0C_2}}{2C_2} \text{ if } c_1 < 0.$$
Hence, there exists a unique non-zero positive real root when $\lambda > 0$ and $\beta_r = 0$.

3. $\lambda = 0$ and $\beta_r > 0$. We rewrite the coefficient c_1 as follows:
$$c_1 = \mu N_r N_h(1 - R_0)(\omega + \gamma_c),$$

where

$$R_0 = R_{0r} + R_{0h} = \frac{\beta_r}{(\omega+\gamma_c)} + \frac{\beta_h \alpha_h}{\mu(\omega+\gamma_c)} \frac{N_r}{N_h}.$$

The polynomial $P(C^*)$ becomes

$$P(C^*) = C_3 C^{*3} \pm C_2 C^{*2} \pm C_1 C^*, \; C_i > 0, i = 1,2,3.$$

Obviously, zero is one of the roots of $P(C^*)$. In case c_2 is negative, we can easily show that c_1 is also negative (see above). Consequently, there exists a unique non-zero positive real root of $P(C^*)$ which is

$$C^* = \frac{-C_2 + \sqrt{C_2^2 + 4C_1C_3}}{2C_3}.$$

If c_2 is positive, c_1 can be either positive or negative. However, if c_1 is positive, the other two roots of $P(C^*)$ are either both real and negative or complex conjugates. There exists a unique non-zero positive real root of $P(C^*)$ if only if c_1 is negative or $R_0 > 1$. Hence, a sufficient condition for a unique non-zero real positive root of $P(C^*)$ is $R_0 > 1$.

4. $\lambda = 0$ and $\beta_r = 0$. The coefficient c_2 is always positive and we write

$$c_1 = \mu N_r N_h (1 - R_{0h})(\omega + \gamma_c).$$

The polynomial $P(C^*)$ can be written in the following form:

$$P(C^*) = C_2 C^{*2} \pm C_1 C^*, \; C_i > 0, i = 1,2.$$

Hence, there exists a unique non-zero positive real root

$$C^* = \frac{C_1}{C_2} \text{ if and only if } c_1 < 0 \text{ or } R_{0h} > 1.$$

In conclusion, for all $\lambda > 0$ and $\beta_r \geq 0$, there always exists a disease-present steady state. In case $\lambda = 0$, for all $\beta_r \geq 0$ there exists the disease-present steady state if and only if $R_0 > 1$.

1.2 Stability analysis

We first study long-term dynamics of (1) when there is admission of colonized residents ($\lambda > 0$). In this case, there only exists a disease-present steady state. The Jacobian matrix of (1) at the disease-present steady state is

$$J^* = \begin{bmatrix} -\lambda(\gamma_u - \gamma_c) + \beta_r - 2\frac{\beta_r}{N_r}C^* - \frac{\beta_h}{N_h}H_c^* - (\omega+\gamma_c) & \frac{\beta_h}{N_h}(N_r - C^*) \\ \frac{\alpha_h}{N_h}(N_h - H_c^*) & -\frac{\alpha_h}{N_h}C^* - \mu \end{bmatrix}.$$

The characteristic equation of J^* is

$$z^2 + a_1 z + a_2 = 0,$$

with

$$\begin{aligned} a_1 &= \mu + \tfrac{\alpha_h}{N_h}C^* + \lambda(\gamma_u - \gamma_c) + (\omega+\gamma_c) + 2\tfrac{\beta_r}{N_r}C^* + \tfrac{\beta_h}{N_h}H_c^* - \beta_r, \\ a_2 &= \left(\mu + \tfrac{\alpha_h}{N_h}C^*\right)\left[\lambda(\gamma_u - \gamma_c) + (\omega+\gamma_c)2\tfrac{\beta_r}{N_r}C^* + \tfrac{\beta_h}{N_h}H_c^* - \beta_r\right] - \tfrac{\beta_h}{N_h}\alpha_h(N_r - C^*)(N_h - H_c^*). \end{aligned}$$

At the disease-present steady state (from the right-hand sides of (1) equaling zeros), we have

$$\lambda(\gamma_u - \gamma_c) + (\omega + \gamma_c) = \frac{1}{C^*}\left[\lambda\gamma_u N_r + \left(\frac{\beta_r}{N_r}C^* + \frac{\beta_h}{N_h}H_c^*\right)(N_r - C^*)\right]$$

and

$$\mu + \frac{\alpha_h}{N_h}C^* = \frac{\alpha_h}{H_c^*}C^*.$$

Substituting these two terms into a_1 and a_2, we obtain

$$a_1 = \mu + \frac{\alpha_h}{N_h}C^* + \lambda\gamma_u\frac{N_r}{C^*} + \frac{\beta_h}{N_h}\frac{H_c^*}{C^*} + \frac{\beta_r}{N_r}C^* + \frac{\beta_h}{N_h}H_c^*$$

and

$$a_2 = \alpha_h\lambda\gamma_u\frac{N_r}{H_c^*} + \alpha_h\frac{\beta_h}{N_h^2}H_c^*(N_r - C^*) + \alpha_h\frac{\beta_r}{N_r}\frac{C^{*2}}{H_c^*} + \alpha_h\frac{\beta_h}{N_h}C^*.$$

Clearly, $a_1 > 0$ and $a_2 > 0$. By the Routh-Hurwitz criteria, the disease-present steady state is stable for all positive C^*. Because we can always find a unique non-zero real positive root of $P(C^*)$ for all $\lambda > 0$ and $\beta_r \geq 0$, the disease-present steady state is stable.

Secondly, let us consider a stability condition of (1) when there is no admission of colonized residents ($\lambda = 0$). When $\lambda = 0$, there are two steady states: the disease-free and present steady states. The disease-free steady state always exists. However, the disease-present steady state exists if and only if $R_0 > 1$. At the disease-free steady state, the Jacobian matrix is

$$J^0 = \begin{bmatrix} \beta_r - (\omega + \gamma_c) & \beta_h\frac{N_r}{N_h} \\ \alpha_h & -\mu \end{bmatrix}.$$

Consequently, we have

$$\text{trace}(J^0) = (\omega + \gamma_c + \mu)\left(\frac{\beta_r}{(\omega + \gamma_c + \mu)} - 1\right), \text{ and } \det(J^0) = \mu(\omega + \gamma_c)(1 - R_0).$$

If $R_0 < 1$, we have $\det(J^0) > 0$, and $\text{trace}(J^0) < 0$ because $\frac{\beta_r}{(\omega+\gamma_c+\mu)} < R_{0r} < 1$. By the Routh-Hurwitz criteria, the disease-free steady state is stable if $R_0 < 1$. In a similar way in deriving a stability condition for the disease-present steady state when $\lambda > 0$, the disease-present steady state is stable if $R_0 > 1$. Therefore, the disease-free steady state is stable if and only if $R_0 < 1$ and the disease-present steady state is stable if and only if $R_0 > 1$.

1.3 Hand hygiene compliance

One of the methods to investigate hand hygiene compliance in mathematical models is by including the term $(1 - \eta)$, where η is the fraction of HCW/resident hand hygiene compliance ($\eta = 0$ means no compliance and $\eta = 1$ means perfect compliance) in the transmission terms (DAgata et al. [1]). This $(1 - \eta)$ term consequently reduces the transmission rate or the probability of colonization when the number of contacts is fixed. However, we do not consider this factor in detail but only demonstrate that hand hygiene compliance that may relate to the probability of colonization in residents may help to reduce MRSA prevalence in nursing homes.

Figure 1A shows that the prevalence of MRSA increases when the probability of colonization in residents by contacting with colonized residents and the number of contacts among them increase. It also increases when the probability of colonization in residents by contacting with contaminated HCWs and the number of contacts between residents and HCWs increase.

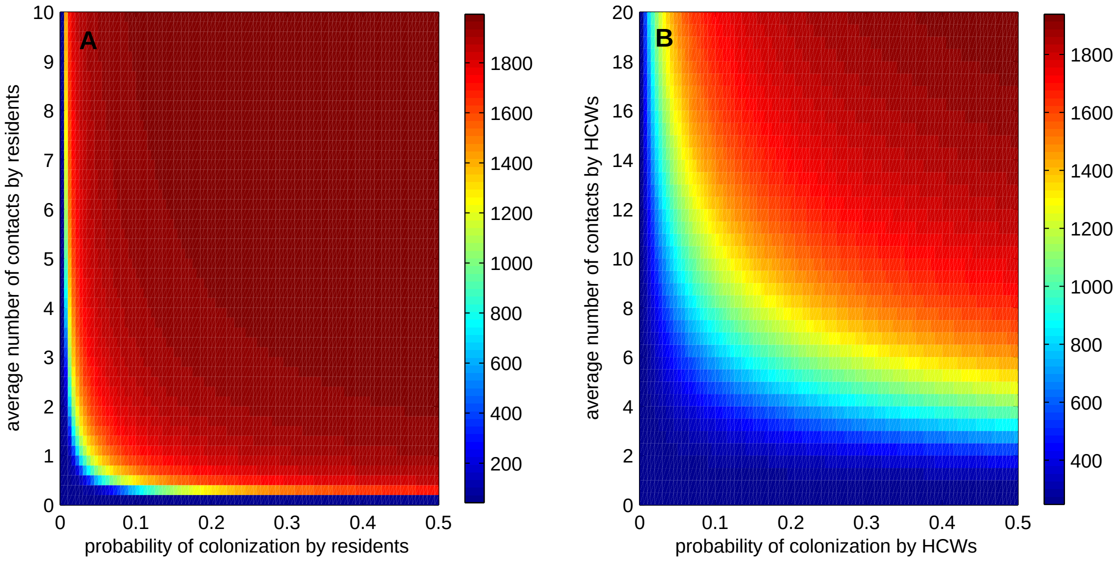

Figure 1: Hand hygiene compliance. (A) Prevalence of MRSA as a function of the average number of contacts among resident and the probability of colonization by contacting with a colonized resident. (B) Prevalence of MRSA as a function of the average number of contacts between HCWs and residents and the probability of colonization by contacting with a contaminated HCW.

2 Derivation of the mean, variance, and covariance equations of the stochastic model

We study a stochastic model based on the ODE system (1) by using the continuous time Markov chain process (CTMC). In the model, time is continuous but state variables are discrete. Because we assume that both of the total populations (residents and HCWs) are constant, the process is bivariate, $\{C(t), H_c(t)\}$ with $U(t) = N_r - C(t)$ and $H(t) = N_h - H_c(t)$. A joint probability function is given by

$$p_{(C,H_c)}(t) = \text{Prob}\{C(t) = C, H_c(t) = H_c\}.$$

This bivariate process has the Markov property and the transition probabilities are shown in Table 2. From transition probabilities, we can write the forward Kolmogorov differential equations as follows:

$$\begin{aligned}
\frac{dp(C,H_c)}{dt} = \; & p(C-1,H_c)\lambda[\gamma_u N_r - (\gamma_u - \gamma_c)(C-1)] \\
& + p(C-1,H_c)\frac{\beta_r}{N_r}(N_r - C + 1)(C-1) \\
& + p(C-1,H_c)\frac{\beta_h}{N_h}(N_r - C + 1)H_c \\
& + p(C+1,H_c)(\omega + \gamma_c)(C+1) \\
& + p(C,H_c-1)\frac{\alpha_h}{N_h}(N_h - H_c + 1)C \\
& + p(C,H_c+1)\mu(H_c+1) - p(C,H_c)\{\lambda[\gamma_u N_p - (\gamma_u - \gamma_c)C] \\
& + \frac{\beta_r}{N_r}(N_r - C)C + \frac{\beta_h}{N_h}(N_r - C)H_c \\
& + (\omega + \gamma_c)C + \frac{\alpha_h}{N_h}(N_h - H_c)C + \mu H_c\}.
\end{aligned}$$

These equations can be used to derive formulae for the rates of change of the expected numbers of colonized residents and contaminated HCWs $(E(C), E(H_c))$, and the higher moments such as

variances and covariance. Here, we introduce the moment generating function (MGF). Define

$$M(\theta_1,\theta_2,t) = M(\theta,t) = E(e^{\theta_1 C+\theta_2 H_c}).$$

We can rewrite the forward Kolmogorov equations in terms of the moment generating function as follows:

$$\begin{aligned}
\tfrac{\partial M}{\partial t} &= (e^{\theta_1}-1)\left(\lambda\gamma_u N_r M - \lambda(\gamma_u-\gamma_c)\tfrac{\partial M}{\partial\theta_1}\right)\\
&\quad +(e^{\theta_1}-1)\left(\beta_r\tfrac{\partial M}{\partial\theta_1} - \tfrac{\beta_r}{N_r}\tfrac{\partial^2 M}{\partial\theta_1^2}\right)\\
&\quad +(e^{\theta_1}-1)\left(\beta_h\tfrac{N_r}{N_h}\tfrac{\partial M}{\partial\theta_2} - \tfrac{\partial^2 M}{\partial\theta_1\partial\theta_2}\right) + (e^{-\theta_1}-1)(\omega+\gamma_c)\tfrac{\partial M}{\partial\theta_1}\\
&\quad +(e^{\theta_2}-1)\left(\alpha_h\tfrac{\partial M}{\partial\theta_1} - \tfrac{\alpha_h}{N_h}\tfrac{\partial^2 M}{\partial\theta_1\theta_2}\right) + (e^{-\theta_2}-1)\mu\tfrac{\partial M}{\partial\theta_2}.
\end{aligned}$$

To derive the equations for means, variances, and covariance of the stochastic model, it is more convenient to use the cumulant generating function which is a logarithm of the moment generating function. Define

$$K(\theta_1,\theta_2,t) = \log M(\theta_1,\theta_2,t).$$

The time derivative for the cumulant generating function K is given by

$$\begin{aligned}
\tfrac{\partial K}{\partial t} &= (e^{\theta_1}-1)\left(\lambda\gamma_u N_p - \lambda(\gamma_u-\gamma_c)\tfrac{\partial K}{\partial\theta_1}\right)\\
&\quad +(e^{\theta_1-1}-1)\left(\beta_r\tfrac{\partial K}{\partial\theta_1} - \tfrac{\beta_r}{N_r}\left[\tfrac{\partial^2 K}{\partial\theta_1^2} + \left(\tfrac{\partial K}{\partial\theta_1}\right)^2\right]\right)\\
&\quad +(e^{\theta_1}-1)\left(\beta_h\tfrac{N_r}{N_h}\tfrac{\partial K}{\partial\theta_2} - \tfrac{\beta_h}{N_h}\left(\tfrac{\partial^2 K}{\partial\theta_1\partial\theta_2} + \tfrac{\partial K}{\partial\theta_1}\tfrac{\partial K}{\partial\theta_2}\right)\right)\\
&\quad +(e^{-\theta_1}-1)(\omega+\gamma_c)\tfrac{\partial K}{\partial\theta_1} + (e^{-\theta_2}-1)\mu\tfrac{\partial K}{\partial\theta_2}\\
&\quad +(e^{\theta_2}-1)\left(\alpha_h\tfrac{\partial K}{\partial\theta_1} - \tfrac{\alpha_h}{N_h}\left(\tfrac{\partial^2 K}{\partial\theta_1\partial\theta_2} + \tfrac{\partial K}{\partial\theta_1}\tfrac{\partial K}{\partial\theta_2}\right)\right).
\end{aligned}$$

The cumulant generating function can be expanded in terms of the cumulants k_{ij} as follows:

$$K(\theta_1,\theta_2,t) = \sum_{k=1}^{\infty}\sum_{j=0}^{k}\frac{k_{j(k-j)}}{j!(k-j)!}\theta_1^j\theta_2^{k-j}.$$

By substituting this power series into the time derivative equation of K, the time evolution of the moments of orders one and two is described by:

$$\begin{aligned}
\tfrac{dE(C)}{dt} &= \lambda[\gamma_u N_r - (\gamma_u-\gamma_c)E(C) + \beta_r E(C) - \tfrac{\beta_r}{N_r}(\mathrm{Var}(C)+E(C)^2)\\
&\quad +\beta_h\tfrac{N_r}{N_h}E(H_c) - \tfrac{\beta_h}{N_h}(E(C)E(H_c)+\mathrm{Cov}(C,H_c)),\\
\tfrac{dE(H_c)}{dt} &= \alpha_h E(C) - \tfrac{\alpha_h}{N_h}(E(C)E(H_c)+\mathrm{Cov}(C,H_c)) - \mu E(H_c),\\
\tfrac{d\mathrm{Var}(C)}{dt} &= \lambda\gamma_u N_r - \lambda(\gamma_u-\gamma_c)(E(C)+2\mathrm{Var}(C)) + 2\beta_r\mathrm{Var}(C)\\
&\quad -2\tfrac{\beta_r}{N_r}T_{CCC} - 4\tfrac{\beta_r}{N_r}E(C)\mathrm{Var}(C) + \beta_r E(C) - \tfrac{\beta_r}{N_r}\mathrm{Var(C)}\\
&\quad -\tfrac{\beta_r}{N_r}E(C)^2 + 2\beta_h\tfrac{N_r}{N_h}\mathrm{Cov}(C,H_c) - 2\tfrac{\beta_h}{N_h}T_{CCH_c}\\
&\quad -2\tfrac{\beta_h}{N_h}(E(C)\mathrm{Cov}(C,H_c) + E(H_c)\mathrm{Var}(C)) + \beta_h\tfrac{N_r}{N_h}E(C)\\
&\quad -\tfrac{\beta_h}{N_h}\mathrm{Cov}(C,H_c) - \tfrac{\beta_h}{N_h}E(C)E(H_c) + (\omega+\gamma_c)(E(C)-2\mathrm{Var}(C)),\\
\tfrac{d\mathrm{Var}(H_c)}{dt} &= 2\alpha_h\mathrm{Cov}(C,H_c) - 2\tfrac{\alpha_h}{N_h}(E(C)\mathrm{Var}(H_c) + E(H_c)\mathrm{Cov}(C,H_c) + T_{CH_cH_c})\\
&\quad +\alpha_h E(C) - \tfrac{\alpha_h}{N_h}\mathrm{Cov}(C,H_c) - \tfrac{\alpha_h}{N_h}E(C)E(H_c) - 2\mu\mathrm{Var}(H_c) + \mu E(H_c),\\
\tfrac{d\mathrm{Cov}(C,H_c)}{dt} &= -\lambda(\gamma_u-\gamma_c)\mathrm{Cov}(C,H_c) + \beta_r\mathrm{Cov}(C,H_c) - \tfrac{\beta_r}{N_r}T_{CCH_c}\\
&\quad -2\tfrac{\beta_r}{N_r}E(C)\mathrm{Cov}(C,H_c) + \beta_h\tfrac{N_r}{N_h}\mathrm{Var}(H_c) - \tfrac{\beta_h}{N_h}T_{CH_cH_c}\\
&\quad -\tfrac{\beta_h}{N_h}(E(C)\mathrm{Var}(H_c) + E(H_c)\mathrm{Cov}(C,H_c))\\
&\quad -(\omega+\gamma_c)\mathrm{Cov}(C,H_c) + \alpha_h\mathrm{Var}(C)\\
&\quad -\tfrac{\alpha_h}{N_h}(E(C)\mathrm{Cov}(C,H_c) + E(H_c)\mathrm{Var}(C) + T_{CCH_c}) - \mu\mathrm{Cov}(C,H_c),
\end{aligned}$$

where T_{CCH_c} is the third central moment $E([C - E(C)]^2[H_c - E(H_c)])$, for example. For simplicity, all the third central moments are approximated by zeros (Kurtz [2,3]).

3 Additional results for the stochastic model

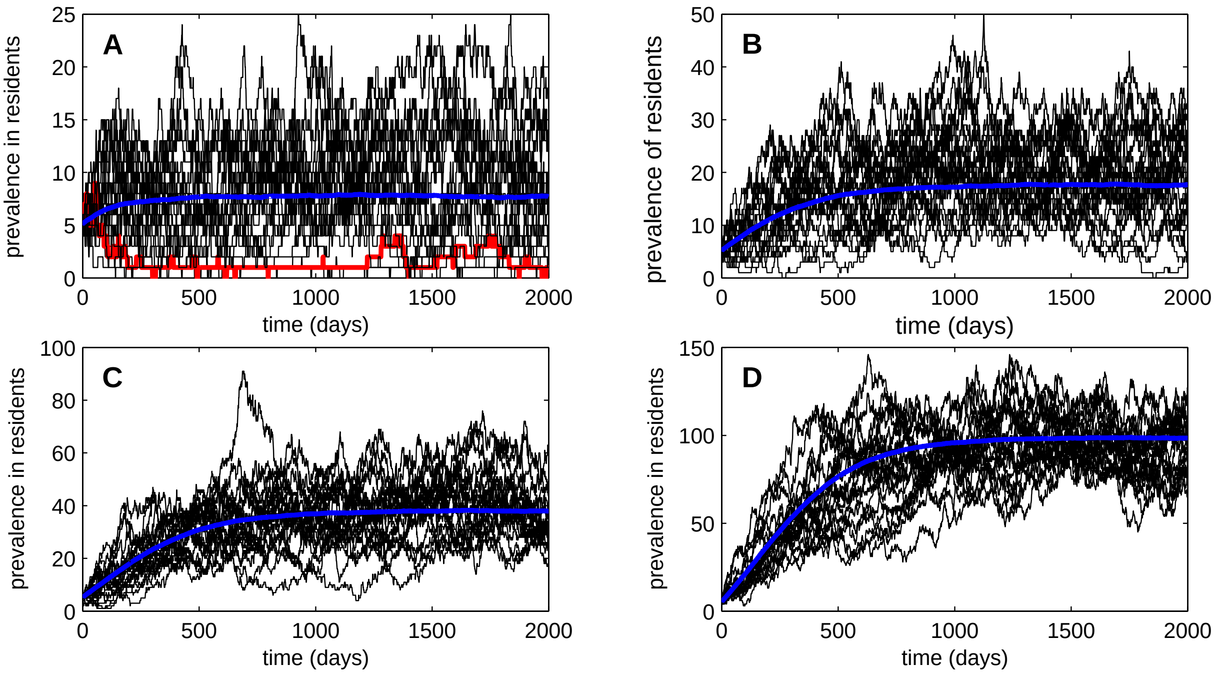

Figure 2: Stochastic results with different sizes of nursing homes. Blue lines represent the mean values of stochastic results evaluated from 5000 realizations. Examples of 20 realizations from 5000 realizations are shown in (A) $N_r = 50$, (B) $N_r = 100$, (C) $N_r = 200$, and (D) $N_r = 500$. Note that the red line in (A) is an example of results when MRSA goes extinct and emerges again. One possibility accounting for this occurrence is the presence of colonized residents at admission.

References

[1] DAgata EMC, Webb G and Horn M (2005) A mathematical model quantifying the impact of antibiotic exposure and other interventions on the endemic prevalence of vancomycin-resistant enterococci. J Infect Dis 192: 2004-2011.

[2] Kurtz TG (1970) Solutions of ordinary differential equations as limits of pure jump Markov processes. J Appl Probab 7: 49-58.

[3] Kurtz TG (1971) Limit theorems for sequences of jump Markov processes approximating ordinary differential equations. J Appl Probab 8: 344-356.
